# ERpS294 is a biomarker of ligand or mutational ERα activation and a breast cancer target for CDK2 inhibition

**DOI:** 10.18632/oncotarget.12735

**Published:** 2016-10-18

**Authors:** Gary K. Scott, David Chu, Ravneet Kaur, Julia Malato, Daniel E. Rothschild, Katya Frazier, Serenella Eppenberger-Castori, Byron Hann, Ben Ho Park, Christopher C. Benz

**Affiliations:** ^1^ Buck Institute for Research on Aging, Novato, CA, USA; ^2^ The Johns Hopkins University School of Medicine, Baltimore, MD, USA; ^3^ Helen Diller Family Comprehensive Cancer Center, University of California, San Francisco, CA, USA; ^4^ Institute for Pathology, Basel University Hospital, Basel, Switzerland

**Keywords:** ERα phosphorylation, *ESR1* mutations, cyclin-dependent kinase-2 inhibitors

## Abstract

ERα phosphorylation at hinge site S294 (pS294) was recently shown to be essential for ER-dependent gene transcription and mediated by an unknown cyclin-dependent kinase (CDK). This study was undertaken to identify the exact CDK pathway mediating pS294 formation, and to determine if this phosphorylation event occurs with, and can be targeted to treat, the ligand-independent growth of breast cancers expressing endocrine-refractory *ESR1* mutations. Using a newly developed anti-pS294 monoclonal antibody, a combination of CDK specific siRNA knockdown studies and a broad panel of CDK selective inhibitors against ligand (E2)-stimulated MCF7 cells, we first identified CDK2 as the primary mediator of pS294 formation and showed that CDK2-selective inhibitors like Dinaciclib, but not CDK4/6 inhibitors like Palbociclib, can selectively prevent pS294 formation and repress ER-dependent gene expression. We then expressed the ER-activating mutations ERmut(Y537S) and ERmut(D538G) in MCF7 cells, and demonstrated their ability to induce ligand-independent and tamoxifen-resistant growth, associated with constitutive and CDK2-dependent pS294 expression. Following robust growth of E2-independent and TAM-resistant MCF7mutER(Y537S) tumors in vivo, nude mice were also treated with either Dinaciclib or Palbociclib at doses and injection schedules unable to retard tumor growth as single agents; the TAM plus Palbociclib combination arrested further tumor growth without affecting pS294 formation, while the TAM plus Dinaciclib combination produced tumor regression associated with loss of pS294 expression. These findings, and our proposed mechanistic model, provide new rationale for the clinical evaluation of CDK2 inhibitors given in combination with endocrine agents as a new treatment strategy against *ESR1* mutation expressing breast cancers.

## INTRODUCTION

Despite estrogen receptor-alpha (ERα) being one of the earliest known and best validated protein targets for cancer therapeutics, our incomplete knowledge about its full molecular structure, mechanism of action, and multiple roles in intracellular signaling and transcriptional control of both normal organ development and malignant tumor growth continues to foster an industry of basic and translational research on this nuclear receptor system [1, 2]. Not least among our knowledge gaps, and prompted largely by the pressing need for more biomarker specificity to predict clinical responsiveness to ER-targeted endocrine agents like antiestrogens (e.g. tamoxifen) and aromatase inhibitors, is understanding the functional role of the many posttranslational modifications (PTMs) now being documented across this ~67 kDa nuclear receptor protein, including its sites of phosphorylation, methylation, acetylation, sumoylation and ubiquitination. Indeed, the overall constellation of PTMs in tumor-expressed ERα may be considered a molecular code reflecting its mode of intracellular receptor activation (e.g. ligand-dependent, ligand-independent) and response to cross-talk, its protein conformation, intracellular localization, and transcriptional competency [3].

ERα phosphorylation, first described over three decades ago, is unquestionably the best studied of all its PTMs, particularly those most commonly observed serine (S) phosphorylation events in ER-positive breast cancer cells detectable by either site-specific antibodies or modern mass spectrometry approaches [4–7]: phosphorylation of the receptor's N-terminal (AF-1) domain at S118 and S167 [8, 9], and its more recently described hinge and ligand-binding domain (LBD) phosphorylation at S294 and S305 [10, 11]. While preclinical evidence indicates that each of these four different phosphorylation events regulate ERα transcriptional activity to some degree, more limited clinical evidence suggests that their prognostic and predictive values are site-specific and not equivalent, with pS118 and pS167 tumor immunoreactivity associated with antiestrogen responsiveness while pS305 tumor immunoreactivity is associated with antiestrogen resistance [8–10, 12, 13]. One reasonable explanation for their different clinical values as individual biomarkers is that each of these ERα phosphorylation events is mediated by a different set of signal-activated kinases [3, 7, 11], with pS118 and pS167 induced by kinases activated during both ligand-dependent and ligand-independent ERα activation (e.g. IKKα, MAPK, S6K1, AKT, and RSK), while pS305 is induced by other kinases associated with ligand-independent activation (e.g. PAK1 and PKA), and pS294 is induced by yet another kinase family associated with ligand-dependent ERα activation (CDK). How these different site-specific serine kinases become recruited to the receptor in response to different types of ERα activating stimuli remains largely unknown. Moreover, with the important recent finding of recurrent LBD hotspot mutations in *ESR1* (encoding ERmut) arising during metastatic progression of endocrine-refractory ER-positive breast tumors [14–16], coupled with structural evidence that these *ESR1* mutations constitutively activate ERmut in a ligand-independent (and ligand-excluding) manner [17, 18], there is no information yet available about the role, if any, receptor phosphorylation may play when breast cancers become driven by *ESR1* mutations like Y537S or D538G.

We previously employed mass spectrometry to detect pS294 expression in various ER-positive breast cancer cell line models subjected to agonistic ligand stimulation [11]; notably, unlike pS118 which can be induced by either ligand or growth factor stimulation, pS294 was shown to be the only phosphorylation site on ERα induced exclusively by ligand binding. Additionally, the mass spectrometry study also demonstrated that pS294 formation is mediated by a serine/threonine protein kinase from the cyclin-dependent kinase (CDK) family [11]; however, we did not know which CDK from that 20-member kinase family actually phosphorylates ERα at that hinge site, a clinically relevant question since CDK4/6-selective inhibitors are now approved and being developed to treat ER-positive breast cancers [19], while other CDK1/2-selective inhibitors are being developed to treat other types of malignancies [20–22]. Therefore, using a newly developed anti-pS294 rabbit monoclonal antibody, the present study was undertaken to identify the exact CDK pathway mediating pS294 induction and to explore the potential role of pS294 formation in driving the ligand-independent growth of breast cancers expressing endocrine-refractory *ESR1* mutations.

## RESULTS

### pS294 immunoreactivity, induction kinetics, and variable expression in ER-positive human breast tumors

**Figure 1:**
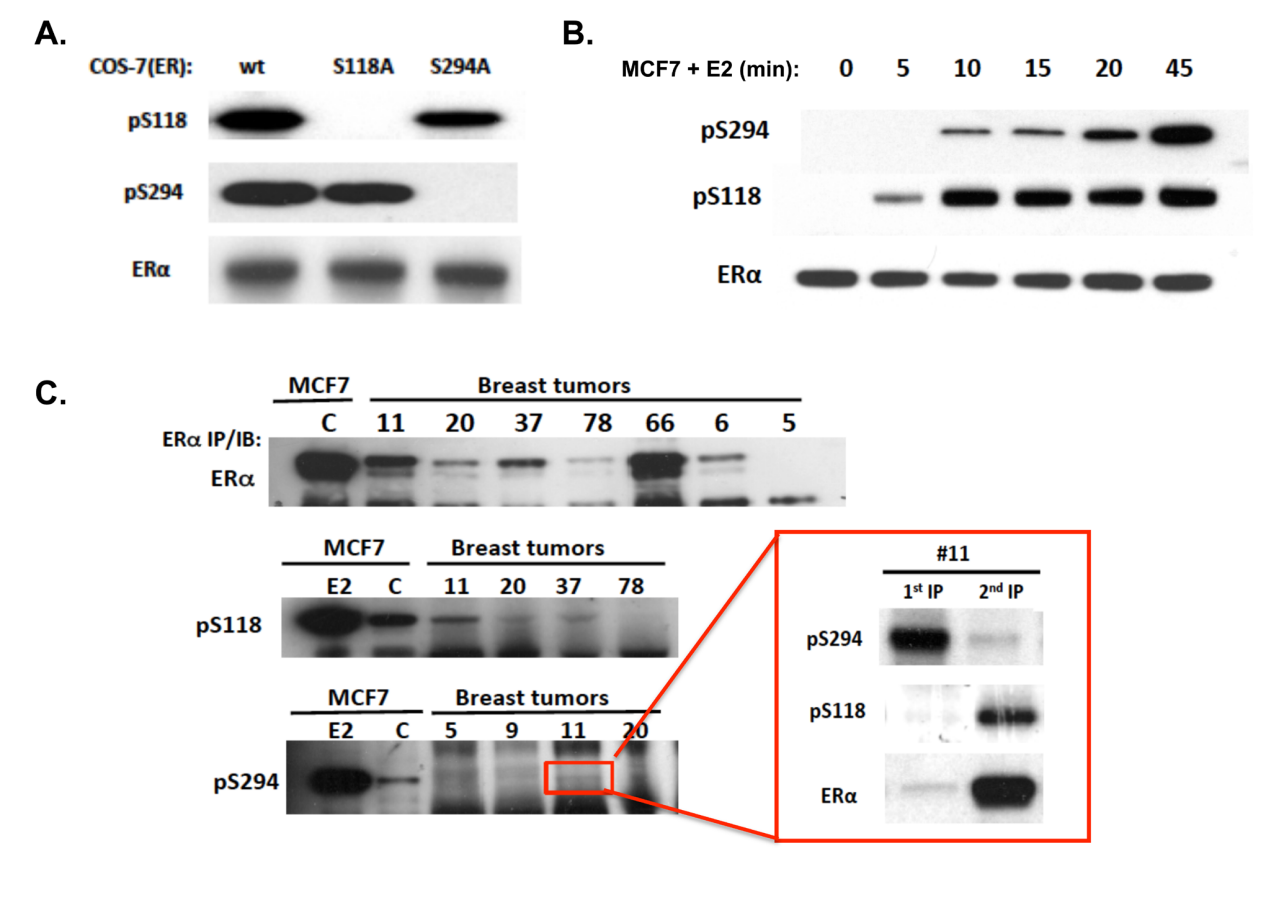
Specific immunoreactivity of pS294, its induction kinetics relative to pS118 in an ER-positive breast cancer cell line (MCF7), and the variable expression of endogenous pS294 found in ER-positive human breast tumors **A.** To demonstrate the immunospecificity of a newly developed anti-ERpS294 rabbit monoclonal, COS-7 cells were transfected with either wildtype (wt) ERα, ERα mutated at S294 (S294A) or ERα mutated at S118 (S118A); 24 h after transfection, media was changed to charcoal-stripped serum containing media for 24 hours, followed by E2 treatment at 10 nM for 45 min, and cells were then lysed, ERα immunoprecipitated and probed by western blotting. **B.** To compare the ligand induction kinetics of pS294 relative to pS118, MCF7 cells grown for >24 h in charcoal-stripped media were treated with E2 (10 nM) for the indicated times, ERα was immunoprecipitated and probed via western blot for pS294, pS118, and total ERα. **C.** To compare detection of pS294 expression in representative ER-positive primary breast tumor samples (#5, 6, 9, 11, 20, 37, 66, 78) relative to control (C) or E2 (45 min) treated MCF7 cells, whole cell lysates were first immunprecipated (IP) for total ERα. and further immunoblotted (IB) for pS294, pS118, and total ERα. As shown in the inset for tumor sample #11, parallel lysate aliquots were first immunoprecipitated for pS294 and that 1^st^ IP immunblotted for pS294, pS118, and total ERα content (1^st^ IP), while the remaining unprecipitated lysate was then immnoprecipitated for total ERα and immunoblotted for pS294, pS118, and total ERα (2^nd^ IP).

To demonstrate the immunospecificity of our newly developed anti-pS294 rabbit monoclonal, COS-7 cells were transfected with either wildtype (wt) ERα, ERα mutated at S294 (S294A), or ERα mutated at S118 (S118A). As shown in Figure 1A, specific pS294 induction is seen 45 min after E2 stimulating the wt or S118A transfected COS-7(ER) cells, but not in stimulated cells transfected with S294A. While detection of endogenously expressed pS294 and pS118 are readily seen by first immunoprecipitating total ERα and then probing the immunoprecipitate for phosphorylated forms of ERα, we have also shown that our rabbit monoclonal can be used to first immunoprecipitate pS294 from cell or tumor lysates (as shown in Figure 1C, inset). Figure 1B shows the ligand induction kinetics of pS294 relative to pS118 in wt MCF7 cells that were grown in estrogen-free media and stimulating with E2 (10 nM) before extracting and analyzing cell lysates (as in Figure 1A) for pS294, pS118, and total ERα. In this model system, while pS118 induction is detectable by 5 min and peaks within 10 min, pS294 induction is more delayed, appearing at 10 min and not peaking for at least 45 min.

To look for evidence of pS294 expression in human breast tumors, we surveyed 18 cryobanked ER-positive breast tumor samples. Given some evidence that pS118 and pS305 tumor expression show opposite clinical associations with antiestrogen responsiveness [8,10], we attempted to compare these phosphorylated forms of ERα with pS294 expression by immunoprecipitating the tumor lysates for total ERα and then western blotting for total and phosphorylated forms of ERα. Two samples failed to show detectable ERα (samples #4, 5); and 3 others showed such low total ERα that we could not clearly detect phosphorylated forms (#6, 20, 78). Figure 1C shows representative results from this tumor survey. From the 13 tumor samples in which some phosphorylated ERα could be detected, pS118 appeared much more prominent than either pS294 or pS305; in 7 of these samples (#11, 14, 22, 33, 39, 69, 146), pS294 expression appeared greater than pS305 while in 6 samples (#9, 13, 26, 37, 46, 66) the reverse was apparent. Given the barely detectable pS294 and pS305 expression by this approach, we re-extracted a few residual tumor samples and first immunoprecipitated them for pS294 (we could not continue our pS305 analysis due to commercial withdrawal of the anti-pS305 monoclonal); this reverse approach proved to be the more sensitive means of assessing pS294 expression in either tumors or cells, as exemplified by tumor sample #11 (inset Figure 1C).

### CDK2 mediates pS294 formation, and CDK2 selective inhibitors repress pS294 and ER-dependent gene expression

**Figure 2:**
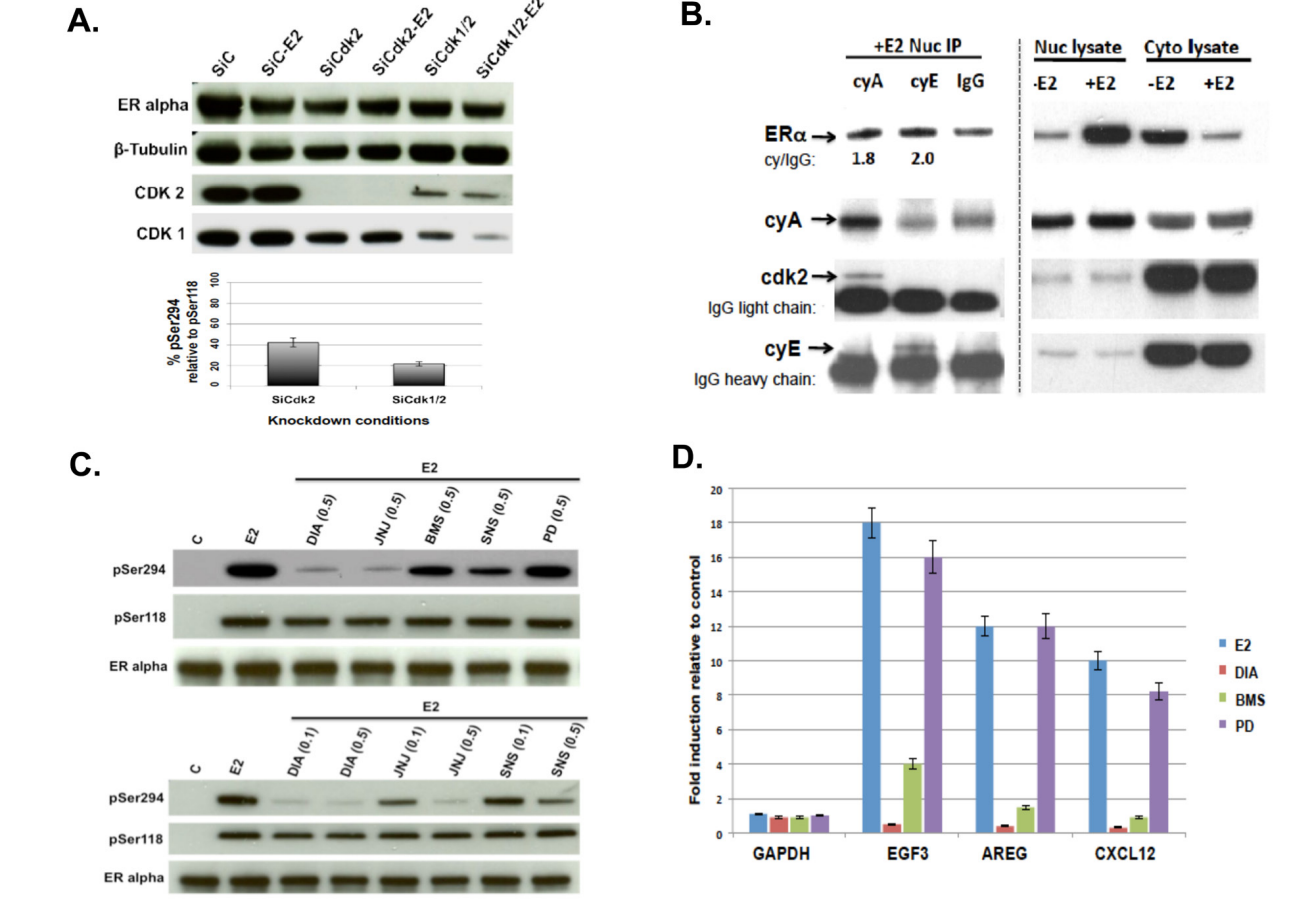
ERα ligand binding triggers rapid association with cyclin A/E-associated CDK2, whose suppression or enzymatic inhibition not only prevents pS294 formation but also the transcription of ERα inducible genes (EGF3, AREG, CXCL12) **A.** Knockdown of CDK1 and/or CDK2 was performed on replicate wells of MCF7 cells transfected with either control (C), CDK2 or CDK1/2 targeted siRNA; 24 h later cultures were changed to phenol red-free media containing 10% charcoal-stripped serum and allowed to grow for another 24 h before treatment with E2 (10 nM x 30 min), followed by cell harvesting, protein extraction and western blotting for ERα, β-tubulin, CDK1 and CDK2 as shown. In parallel, immunoprecipitation of total ERα from the cell lysates was followed by western blotting to detect pS294 and pS118 levels; and densitometry measured band intensities were used to quantitate pS294 levels relative to pS118 levels after knockdown of CDK2 alone (SiCdk2) or combined CDK1/2 knockdown (SiCdk1/2) under E2 exposure, relative to control siRNA (SiC-E2) treatment conditions. The average relative declines (*n* = 3, SEM) in pS294 (relative to pS118) are shown in the bar graphs below. **B.** MCF7 cells grown in charcoal stripped and phenol red free media were treated +/- ligand (-E2 or +E2, 10 nM x 30 min), gently lysed and nuclei pelleted and extracted, producing cell fractions (Nuc lysate, Cyto lysate) that were immunoblotted for ERα, cyclin A2 (cyA), cyclin E (cyE) or CKD2 as shown. In parallel, the E2 treated Nuc lysates were first immunoprecipitated using anti-cyA, anti-cyE, or control anti-IgG and then immunoblotted for ERα, cyA, cyE and CKD2. **C.** MCF7 cultures pretreated for 60 min with either vehicle (C) or the indicated dose (μM) of CDK inhibitor (DIA = Dinaciclib, JNJ = JNJ7706621, BMS = BMS265246, SNS = SNS-032, PD = PD0332991/Palbociclib) were then stimulated for 20 min with E2 before harvest, protein extraction, ERα immunoprecipitation, and immunoblotting for pS294, pS118, and total ERα as shown. **D.** MCF7 cells grown in charcoal stripped and phenol red-free media were pretreated for 1 h with 0.5 μM DIA , 1 μM PD, or 1 μM BMS followed by 6 h E2 (10 nM) treatment, after which total RNA was extracted and semiquantitative RT-PCR performed to measure fold induction of the ERα inducible genes EGF3, AREG, and CXCL12 relative to the housekeeping gene GAPDH, using previously described primers and methods [11].

To identify the exact CDK pathway mediating pS294 formation and determine if this could be a therapeutic target, we first employed CDK-specific siRNA knockdown studies and then tested a broad panel of CDK selective inhibitors against ligand (E2)-stimulated MCF7 cells. As shown in Figure 2A, complete knockdown of CDK2 was well tolerated and resulted in 58% reduction in E2 induced pS294 relative to pS118 expression. CDK1 knockdown was quickly lethal to the MCF7 cells; but, since CDK1 can compensate for loss of CDK2, we attempted to evaluating combined partial knockdown of both mitotic CDKs, CDK1/2, which produced a 78% reduction in ligand induced pS294 relative to pS118. In contrast, knockdown of the non-mitotic CDK9, an additional target of some of the CDK inhibitors studied, had absolutely no impact on E2 induction of pS294 in this same cell system (Supplement Figure S1). Since CDK2 is mitotically activated by cyclins A and E, we looked for the co-association of these cyclins with nuclear ERα immunoprecipitated after E2 induction of MCF7 cells (Figure 2B). E2 induced nuclear localization of ERα as expected; however, separation of nuclei from the cytoplasmic fractions left most of the cyclin E in the latter, presumably not bound to a large enough complex to avoid leaking from the nuclei in these asynchronously growing cells. In contrast, most cyclin A remained in the nuclei and was not only enriched in the nuclear ERα immunoprecipitates but also co-associated with CDK2 (Figure 2B).

To further discriminate between CDK1 and CDK2 loss of function with regard to pS294 formation and to compare with loss of CDK4/6 function, we tested the broad panel of CDK selective inhibitors described in Table 1. As shown in Figure 2C, the ability of four different CDK inhibitors prevented pS294 formation in rank order according to their CDK2 targeting potency (IC_50_ values): Dinaciclib > JNJ-7706621 > SNS-032 and BMS-265246, which also appeared independent of their CDK1 selectivity since SNS-032 is at least 10-fold more potent against CDK2 than CDK1 [23]. To confirm the preferred dependence of pS294 on CDK2 rather than CDK1, a potent new CDK2 inhibitor >100-fold more selective for CDK2 than CDK1, CYC065 [24], was shown to prevent pS294 formation almost as efficiently as Dinaciclib in MCF7 and SUM44 cells (Supplement Figure S2). Of note, the clinically approved CDK4/6 inhibitor Palbociclib had no ability to prevent pS294 formation (Figure 2C). Also notable, the potent mitogen activated protein kinase (MAPK) p38 inhibitor, SB203580, is unable to prevent pS294 induction (Supplement Figure S2). Evaluation the same three ER-dependent gene transcripts (EGF3, AREG, CXCL12) that we first showed could be inhibited by mutating the ERα S294 site to prevent its phosphorylation [11], we now show that E2 induction of these genes in MCF7 cells can be prevented by the CDK2 inhibitors, Dinaciclib and BMS-265246, but not by the CDK4/6 inhibitor Palbociclib (Figure 2D). These same CDK2 inhibitors (Dinaciclib, BMS-265246), but not the CDK4/6 inhibitor (Palbociclib), are able to cooperate with tamoxifen to induce apoptosis in MCF7 cells (Supplement Figure S3).

**Table 1:** CDK inhibitors and their target specificities, evaluated in this study

CDK Inhibitors	Target Selectivity (IC_50_)*
Dinaciclib/SCH-727965	CDK1 (3 nM), CDK2 (1 nM), CDK5 (1 nM), CDK9 (4 NM)
SNS-032	CDK2 (38 nM), CDK7 (62 nM), CDK9 (4 nM)
BMS-265246	CDK1 (6 nM), CDK2 (9 nM)
JNJ-7706621	CDK1 (9 nM), CDK2 (4 nM)
CYC065	CDK2 (5 nM), CDK9 (26 nM)
Palbociclib/PD-0332991	CDK4 (11 nM), CDK6 (16 nM)

*Half maximal inhibitory concentrations (IC_50_) of drug assayed in cell-free systems; all values from Selleckchem.com, except for CYC065 [24].

**Figure 3:**
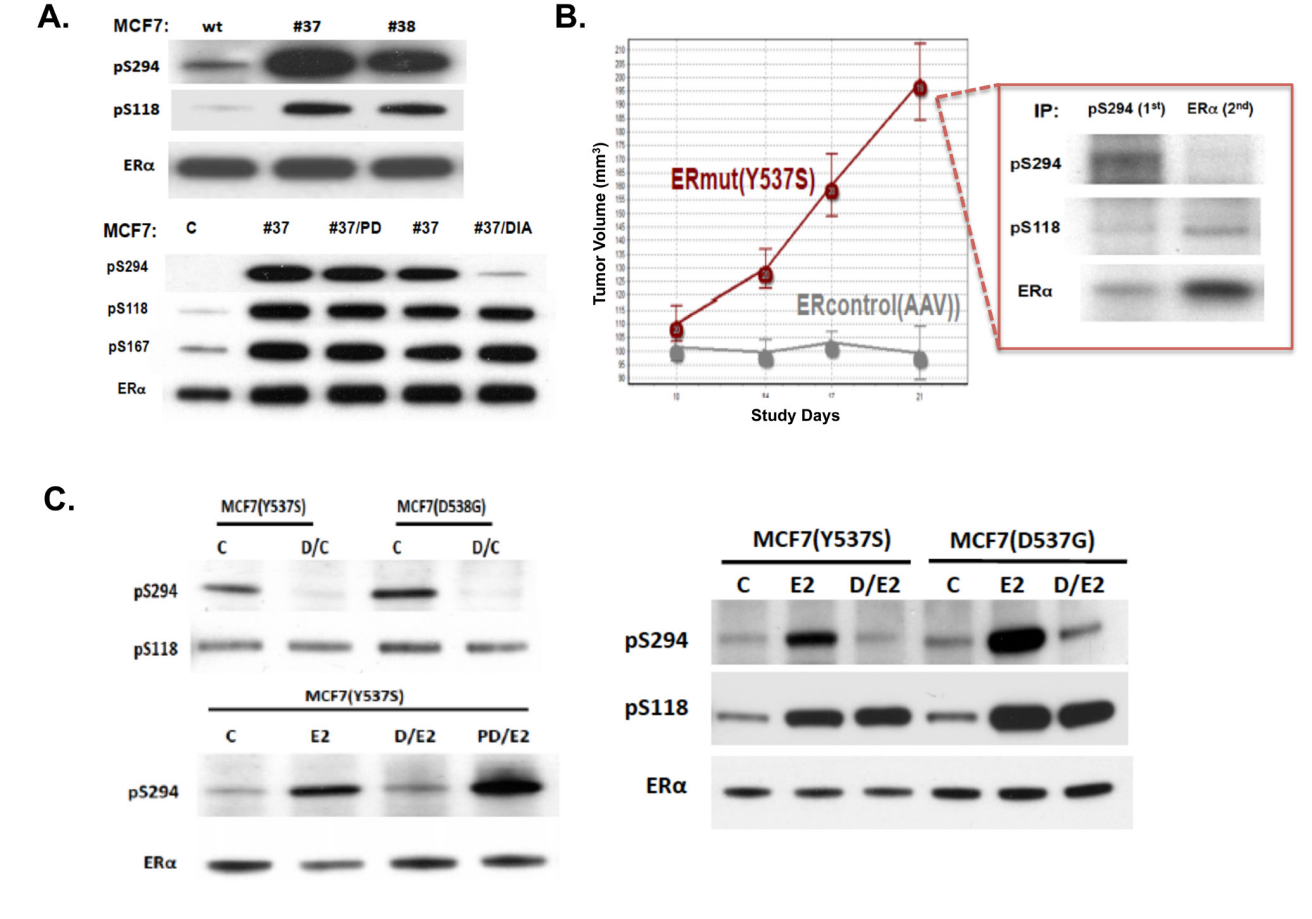
Transient and constitutive MCF7 overexpression of Y537S or D538G mutated ERα (mutER) induces ligand-independent tumorigenic growth with pS294 formation, prevented by the CDK2 inhibitor Dinaciclib but not by the CDK4/6 inhibitor Palbociclib **A.** Following transient transfection of MCF7 cells with control (C or wt = pSG5-HEGO) or mutER expression vectors (#37 = Y537S, #38 = D538G), transfer to E2-free media (x 24-48 h), and subsequent culture treatment (0.5 μM x 60 min) with either Dinaciclib (DIA) or Palbociclib (PD), cells were harvested, proteins extracted, ERα immunoprecipitated and immunoblotted for pS294, pS118, pS167, and total ERα. **B.** Young nude mice were bilaterally implanted with knock-in MCF7 sublines (8 mice per subline), ERmut(Y537S) or ERcontrol(AAV), and observed for tumorigenic growth in the absence of exogenous E2 supplementation, with only the ERmut expressing cells showing E2-independent tumor growth. At 21 days, tumors were excised, protein extracted, immunoprecipitated first for pS294 (1^st^ IP) and then for remaining ERα (2^nd^ IP). Both IPs were immunoblotted for pS294, pS118, and total ERα. **C.** Knock-in MCF7 sublines, MCF7(Y537S), MCF7(D538G) and control MCF7(AAV), were serially passaged in E2 free media (C), transiently stimulated with E2 (10 nM x 20 min) +/- pretreatment (0.5 μM x 60 min) with Dinaciclib (D) or Palbociclib (PD) before cell harvest, protein extraction, ERα immunoprecipitation and immunoblotting for pS294, pS118, and total ERα as shown.

### ESR1 mutations induce ligand-independent and tamoxifen-resistant tumor growth with CDK2-dependent pS294 expression

MCF7 expressing ERα activating mutations (Y537S, D538G) were produced by either transient transfection (Figure 3A) or knock-in (Figure 3B, 3C), and these sublines were evaluated for their ability to produce pS294 in the absence of ligand stimulation and for their dependence on CDK2. When expressed in parental MCF7 cells, both sets of activating mutations caused ligand-independent induction of pS118, pS167 and pS294, with only the latter being preventable by Dinaciclib, and none of these ERα phosphorylation events preventable by Palbociclib (Figure 3A, 3C). When the knock-in sublines, MCF7mutER(Y537S) and MCF7controlER(AAV), were inoculated into immunocompromised mice (not supplemented with E2) only the MCF7mutER(Y537S) subline showed ligand-independent tumor formation, and these tumors showed robust growth rates (Figure 3B) associated with constitutive formation of both pS294 and pS118 (Figure 3B inset).

**Figure 4:**
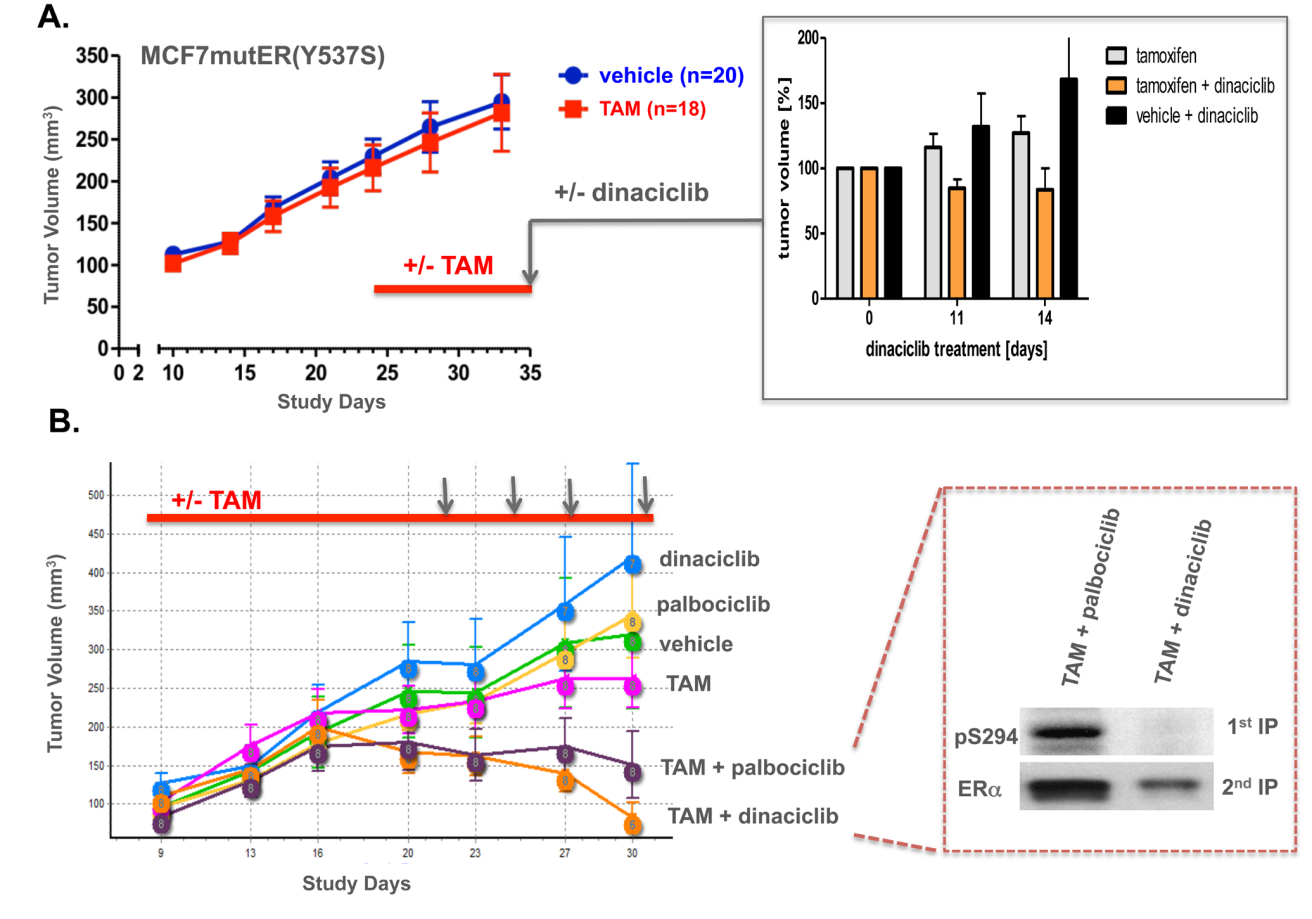
E2-independent MCF7mutER(Y537S) tumor xenografts are resistant to tamoxifen (TAM) but regress when TAM is combined with Dinaciclib, given at a dose and schedule that alone has no effect on tumor growth **A.** In study PTC1797, MCF7mutER(Y537S) implanted nude mice showing tumor growth in the absence of exogenous E2 supplementation were allocated to begin treatment at day 24 with either daily sc 0.5 mg tamoxifen citrate (18 mice, red bar) or vehicle (20 mice). At day 35, mice with comparably sized tumors from each group were also begun with ip treatments receiving either vehicle or Dinaciclib (30 mg/kg) twice weekly over the next 14 days. Inset shows % tumor volume change beyond day 35 for TAM (*n* = 8), Dinaciclib (n = 6), and TAM + Dinaciclib (*n* = 7) treated tumors. **B.** In study PTC1854, MCF7mutER(Y537S) implanted nude mice (without E2 supplementation) showing tumor growth at day 8 were randomized into 6 treatment arms (8 mice/arm) to receive sc daily TAM (*n* = 18, red bar) or vehicle (*n* = 18) as in PTC1797, and at day 22 to begin ip treatments with vehicle (n = 8) or 30 mg/kg of either Palbociclib or Dinaciclib as shown (vertical arrows). Inset shows representative IP/IB analysis of combination treated tumors harvested 2 h following final ip treatment, 1^st^ IP/IB for pS294 and 2^nd^ IP/IB for total ERα.

In culture and in the absence of estrogen, these same sublines not only showed vastly different ligand-independent growth rates, but the more rapidly growing MCF7mutER(Y537S) cells were also minimally affected by Tamoxifen (TAM), while the much slower growing MCF7controlER(AAV) cells were reduced in number by TAM to below their initial culture inoculation density (Supplement Figure S4). To confirm its TAM resistance, the MCF7mutER(Y537S) subline was implanted subcutaneously into 38 nude mice (without E2 supplementation) in our *in vivo* study PTC1797, wherein 24 days following implantation 18 of the tumor bearing mice began daily TAM injections (0.5mg sc) while 20 others received vehicle only injections. After 11 days of this treatment (day 35), there was no significant difference in the mean (+/- SEM) tumor volumes between the treatment arms, confirming the *in vivo* TAM resistance of this MCF7mutER(Y537S) subline. At this time point, 15 of the TAM treated mice and 6 of the control mice were selected to receive an additional 14 days of treatment as follows: continued daily TAM (*n* = 8), 30 mg/kg twice weekly ip Dinaciclib injections only (*n* = 6), or continued TAM plus twice weekly ip Dinaciclib (*n* = 7). As shown in Figure 4A (inset), the TAM only and Dinaciclib only treated groups continued to show growth increases in their tumor volumes, while the TAM plus Dinaciclib treated mice showed an average 20% regression from their day 35 tumor volumes.

Following these PTC1797 findings, a more definitive 6-arm PTC1854 xenograft study was undertaken with the MCF7mutER(Y537S) subline again implanted into nude mice without E2 supplementation (Figure 4B). Daily TAM injections were started for half the group beginning at day 9 while the other half received daily sc vehicle injections; at day 22, twice weekly ip treatments were begun for the 6 groups (*n* = 8 mice/group), each receiving vehicle, 30 mg/kg of either Palbociclib or Dinaciclib, in addition to their daily (TAM or control) sc injections. At study day 31 and within 2-3 h of receiving their 4^th^ and final ip injection, animals were sacrificed and tumors resected and snap frozen for analysis. Despite considerable mouse-to-mouse variation in tumor volumes within each of the 6 treatment arms, PTC1854 confirmed the findings of PTC1797 by showing no significant differences in average final tumor volumes for TAM and control/vehicle-only treated mice, and no tumor inhibiting impact from either single agent Dinaciclib or Palbociclib. Remarkably, the combination treatments either arrested further tumor growth (TAM plus Palbociclib) or caused tumor volume regressions (TAM plus Dinaciclib), indicating a synergistic anti-tumor effect for the combination of agents that by themselves were unable to slow tumor growth (Figure 4B). To establish that the TAM treatments were having some biological effect on the tumors (including their stroma) despite the TAM resistance of MCF7mutER(Y537S) tumor cells, we showed that the TAM injections caused an upregulation in tumor TGFβ-1 and VEGF-A protein expression (Supplement Figure S5), known to be part of an ER-independent TAM response [25–27]. Finally, to differentiate tumor responses to the TAM plus Palbociclib and TAM plus Dinaciclib treatment combinations, we showed that the latter were associated with loss of pS294 tumor expression not seen with the former treatment combination (Figure 4B inset).

**Figure 5:**
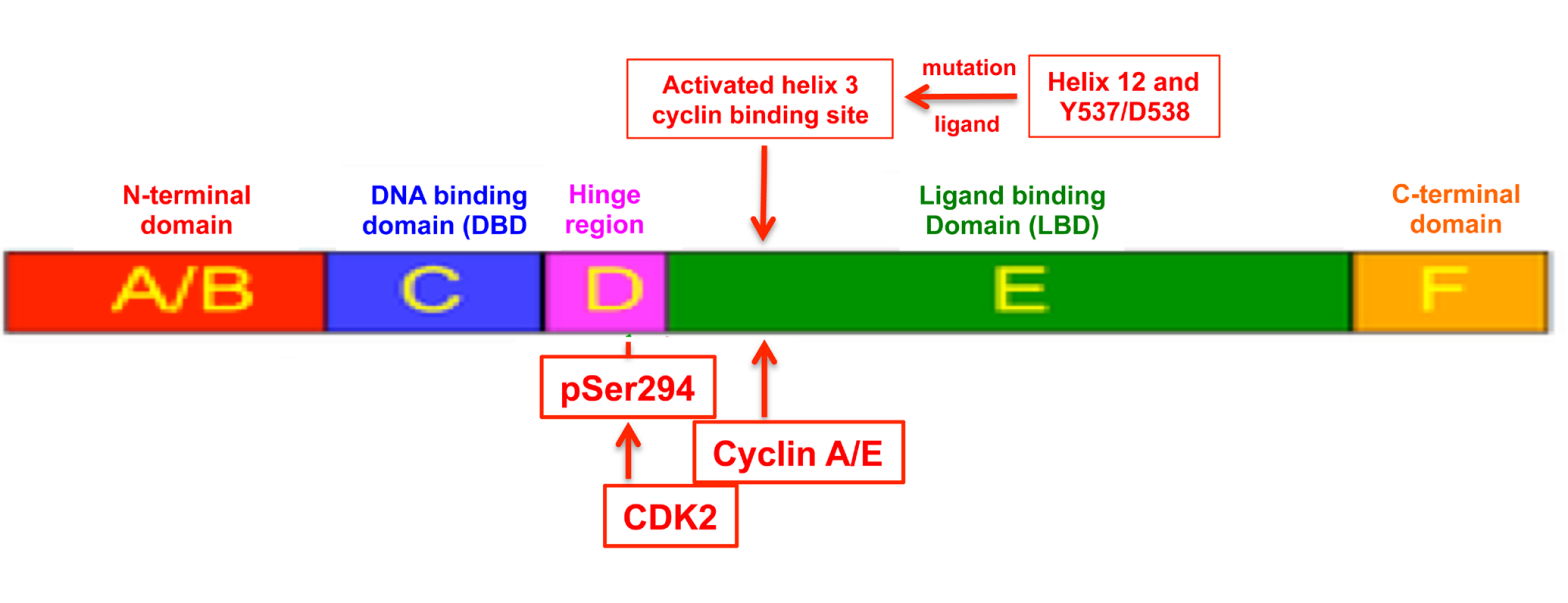
Schematic of proposed mechanism by which activation of the ER ligand binding domain (LBD) recruits cyclin A/E and CDK2 to phosphorylate its hinge S294 site The most recent structural studies predict similarities in the repositioning of helix 12 in proximity to helix 3 [48], induced by either agonist binding within the LBD pocket or by acquisition of LBD mutations like Y537S and D538G in the absence of any ligand binding. We postulate that these LBD structural rearrangements may facilitate cyclin A/E binding to a highly conserved helix-3 RXL site (352-354), enabling receptor recruitment of CDK2 and its subsequent hinge phosphorylation at S294.

## DISCUSSION

Following our mass spectrometry discovery that the ERα hinge site at S294 is rapidly phosphorylated following ligand (E2, TAM) stimulation in a variety of ER-positive breast cancer cell lines [11], we developed a rabbit monoclonal antibody specific for pS294 that now makes available a powerful and facile analytical tool to interrogate endogenous induction of pS294 in relation to other intracellular ERα phosphorylation sites. Using this new immunoreagent we confirmed that pS294 expression is detectable in primary human breast tumors, albeit at seemingly lower levels than ERα AF-1 domain phosphorylation at S118. Unfortunately, the commercial withdrawal of the previously available anti-pS305 monoclonal (clone 124.9.4 from EMD Millipore) thwarted further validation of our earlier conclusion that ligand-activated pS294 induction and growth factor activated pS305 induction are usually mutually exclusive ERα phosphorylation events [11]. However, employing other commercially available and well validated anti-pS118 and anti-pS167 monoclonals to interrogate breast cancer cell line models expressing wildtype ERα, pS294 formation appears to be mechanistically independent from phosphorylation at these other sites in terms of kinetics, mode of receptor activation, and the kinase family mediating this phosphorylation. Of note, and unique to this hinge phosphorylation site, is our immuno-verification that pS294 formation is induced exclusively by agonistic ERα ligand binding and mediated by a CDK family member different from all other kinases promoting ERα phosphorylation in response to ligand or growth factor stimulation.

Our present studies also support previous contentions that the ERα hinge region [28] and its phosphorylation at S294 [29] exert functional significance beyond their role in enabling full transcriptional execution of ER-dependent genes, namely, regulating the poorly understood mechanism of transcription-dependent ER degradation via the proteasomal pathway [30]. In experimental models, mutation at this S294 hinge site or enzymatic inhibition of its phosphorylation can increase intracellular ERα half-life and impair its ligand-activated proteasomal degradation within the nucleus [29]. Despite the efficient immunoprecipitating capacity of our anti-pS294 monoclonal, we have been unable to detect by chromatin immunoprecipitation (even following pretreatment with a proteasome inhibitor like MG-132) DNA-binding of pS294 upon E2 stimulation at E2 inducible gene loci whereas increased DNA-binding by ERα and pS118 following ligand stimulation are readily detectable at these loci (data not shown). These findings suggest a testable new hypothesis that ligand activated and DNA-bound ERα is first removed from the chromatin before being phosphorylated at S294, which tags the cistrome-removed receptor complex for reaction with an E3-ubiquitin ligase like SCF^Skp2^ for its ubiquitination and nuclear degradation [29, 30]. In this regard, we now question earlier conclusions that pS294 formation results from the specific MAPK, p38 [29, 30]. Rather, our studies support bioinformatic predictions that this hinge site is a CDK phosphorylation site; and our combination of CDK isoform-specific siRNA knock-down studies and comparison of a broad panel of CDK isoform-selective inhibitors indicate that CDK2 is the primary mediator of pS294 formation (Figure 2A, 2C). Although we have ruled out p38 involvement as a direct enzymatic mediator of pS294 induction (Supplement Figure S2A), studies in other cell systems showing that p38 activation can induce cyclin A2 leave open the possibility that extended p38 inhibitor treatment could ultimately inhibit CDK2 activity and pS294 formation by reducing cyclin A2 levels [31].

We also present a new model proposing that cyclin-dependent recruitment of CDK2 to the structurally activated domain E (LBD) of ERα enables pS294 formation (Figure 5). While other AF-1 domain phosphorylation events have been shown to occur by kinase recruitment via receptor coactivators like AIB1/SRC-3 [32] or by LBD residues that can directly dock to a specific kinase [33], our findings are the first to suggest that hinge phosphorylation at S294 first depends upon ligand binding or a mutation-associated structural change within the LBD, enabling cyclin A2 and/or E1 to bind to the highly conserved R^352^X^353^L^354^ site (352-354) in helix 3, and thereby recruit and activate CDK2 for its phosphorylation of the adjacent hinge S294 site. It has long been recognized that cyclins can exert unexpected stimulatory and inhibitory functions, dependent or independent of their partner CDKs, on almost all steroid receptor family members, linking their transcriptional activities to cell cycle control and proliferation [34]. Unfortunately, such data with regard to ERα has so far been either sparse or mystifying. In one study, cyclin E was shown to influence the activities of AR and GR, but had no apparent effect on either ERα or PR [35]. In others, cyclin A in concert with CDK2 were reported to potentiate the activity of PR and ER, but by different mechanisms [36, 37]. Potentially relevant to our model, cyclin A was shown to potentiate the ligand-independent activity of ER as well as enhance its tamoxifen induced activity [36]. While seemingly consistent with our model, this latter study was performed before the discovery of S294 phosphorylation and ascribed the cyclin A/CDK2 potentiation of ERα to phosphorylation at S104/S106 [36]. We have not only shown that a potent pan-CDK inhibitor capable of preventing S294 phosphorylation has no effect on MCF7 phosphorylation at S104/S106 [11], these particular AF-1 sites are now thought to be primarily phosphorylated by MAPK and GSK-3 kinases [38, 39]. With our present understanding of the biological importance of pS294 formation under exclusive mechanistic control by CDK2, innovative breast cancer clinical opportunities become apparent given the therapeutic development of increasingly more specific CDK inhibitors [19–22] and the important recent findings of LBD hotspot mutations in *ESR1* arising during breast cancer metastatic progression and driving clinical resistance to both aromatase inhibitors and antiestrogens like tamoxifen [14–18].

Alongside recent approval and growing clinical use of CDK4/6 inhibitors like Palbociclib in combination with endocrine agents to treat metastatic ER-positive breast cancers, there have been parallel advancements in the clinical development of CDK2-selective inhibitors like Dinaciclib (Merck) and CYC065 (Cyclacel) for the treatment of hematopoietic and MYC-activated malignancies and potentially also breast cancers [19–22]. Armed with our structural model that ligand-activated ER recruits CDK2 for its phosphorylation at S294 (Figure 5), and given recent preclinical and clinical observations indicating that when ERα LBD helix 12 mutations occur at hotspot sites like Y537 (e.g. Y537S) and D538 (e.g. D538G) the expressed ERmut is constitutively activated in both a ligand-independent and ligand-excluding manner that can drive hormone-resistant metastatic progression of breast cancers, we predicted that ERmut expressing breast cancers would not only exhibit ligand-independent pS294 expression but also a susceptibility to CDK2 inhibitors not seen with CDK4/6 inhibitors. To address this prediction we developed two different isogenic experimental models of ERmut expressing breast cancers: transient transfection as well as gene knock-in with constitutive expression of either ERmut(Y537S) or ERmut(D538G) within ER-positive MCF7 breast cancer cells. Using these engineered models, we observed *in vitro* and *in vivo* that expression of either ERmut(Y537S) or ERmut(D538G), even in the presence of background wt ERα expression, produced ligand-independent and tamoxifen-resistant breast cancer growth (Figure 3, Supplement Figure S4). As well, we observed that this dysregulated ER-dependent growth was associated with constitutive pS294 expression that could be inhibited by a CDK2 inhibitor like Dinaciclib, but not by a CDK4/6 inhibitor like Palbociclib (Figures 3, 4).

In one particularly informative *in vivo* study (PTC1854) using the MCF7mutER(Y537S) model where E2-independent and TAM-resistant tumor growth was again observed, mice with well established tumors in addition to receiving daily tamoxifen also received parenteral injections of either Dinaciclib or Palbociclib, at equipotent doses previously shown to be well tolerated but unable as single agents to arrest growth of this tumor model (Figure 4B). Remarkably, both of these CDK inhibitors exhibited some degree of anti-tumor synergy in combination with TAM. The slightly greater anti-tumor effect apparent following TAM plus Dinaciclib was associated with loss of pS294 tumor expression, not seen in tumors given the TAM plus Palbociclib combination (Figure 4B). The potential for these two classes of CDK inhibitors to differentially interact with TAM may be due to their very different anti-mitotic mechanisms and phosphorylated substrates, as exemplified in our cell culture results on parental MCF7 cells wherein TAM plus Dinaciclib resulted in a marked degree of apoptosis not seen with the TAM plus Palbociclib combination (Supplement Figure S3). However, the more striking and potentially clinically relevant observation was the unexpected ability of TAM to induce an augmented or synergistic antitumor response to either type of small molecule CDK inhibitor. Addressing this interesting question of how TAM treatment, itself unable to arrest MCF7mutER(Y537S) tumor growth, when given in combination with a seemingly ineffective CDK inhibitor treatment regimen could result in tumor growth arrest or regression, we looked for evidence of an earlier reported TAM effect on ER-negative cells and tumors [25]. In keeping with those prior reports [26, 27], we observed that while the MCF7mutER(Y537S) tumors were not significantly growth arrested by TAM, they were clearly affected by the daily TAM treatments evidenced by their upregulated expression of TGFβ-1 (stromal secreted isoform of TGFβ) and VEGF-A (stromal vascular endothelial growth factor A) (Supplement Figure S5). Presently, we can only postulate that these stromal and/or vascular effects of TAM altered the xenograft tumor physiology in such a way as to improve the intratumor bioavailability and efficacy of Dinaciclib and Palbociclib, enabling their respective CDK inhibiting differences to be seen in terms of tumor regression and pS294 expression. While observed only to date in our MCF7mutER(y537S) experimental model, future studies are needed to confirm that this synergistic phenomenon might also apply to all ER-positive breast tumors, whether or not they express *ESR1* mutations.

In sum, our findings lead us to conclude that pS294 induction is essential for ER-dependent gene transcription and serves as a unique biomarker for both agonistic ligand and mutationally activated tumor ERα. This induction and expression of pS294 in wt ER-positive or ERmut-positive breast cancers can be prevented by CDK2 inhibitors, but not by CDK4/6 inhibitors. While CDK4/6 inhibitors like Palbociclib in combination with TAM may potentially arrest the *in vivo* growth of ERmut expressing breast tumors, CDK2 inhibitors like Dinaciclib when given in combination with TAM may be more effective by inducing regressions in established ERmut expressing tumors. Altogether, these findings support further clinical evaluation of CDK2 inhibitors like Dinaciclib administered in combination with endocrine therapy as a potentially new treatment strategy against *ESR1* mutation expressing breast cancers.

## MATERIALS AND METHODS

### Reagents, antibodies, tumors, cells, and cell viability assay

Under multi-institutional review board approval, 18 cryobanked samples (-80 °C, <100 mg wet weight, archived prior to 1999) from primary invasive breast tumors were provided by the Stiftung Tumorbank Basel (STB, now integrated within the pathology biobank of the University Hospital of Basel), all selected for their ER-positivity based on prior quantitative immunoassay (ER range: 47-387 fmol/mg protein). The human breast cancer cell line MCF7 was obtained from American Type Culture Collection (ATCC) and propagated under recommended conditions: 37°C, 5% CO_2_ in Dulbecco's Modified Eagle's Medium (DMEM) supplemented with 5% fetal bovine serum (FBS) and 1% Penicillin-Streptomycin (Life Technologies). The ER-positive human breast cancer cell line, SUM44, was provided by Stephen Ethier and passaged as previously described [40]. Phenol red-free media supplemented with L-glutamine was obtained from Invitrogen; charcoal stripped serum (CSS) from Hyclone (Thermo Scientific); beta-estradiol (E2) and 4-hydroxytamoxifen (TAM) from Sigma-Aldrich. Cell viability assays were performed in multiwall white walled culture plates by CellTiter-Glo (Promega, Madison, WI) assay, as previously described [41]. ERα mouse monoclonal (clone F-10) and rabbit polyclonal (sc-7207) antibodies were from Santa Cruz Biotechnology; pS118 and pS167 monoclonal antibodies were from Cell Signaling. A pS305 monoclonal antibody (clone 124.9.4), originally obtained from EMD Millipore, became commercially unavailable shortly after study initiation. The newly described rabbit monoclonal antibody to pSer294, was developed in collaboration with Epitomics/Abcam (Burlingame, CA) using splenocyte clones (#37-7 and #65-3) derived from the same peptide-inoculated rabbits used to produce our previously described anti-pS294 antisera [11]. Other immunoprecipitating (IP)/immunoblotting (IB) antibodies were commercially obtained as follows: anti-cyclin A2, anti-cyclin E1 and anti-TGFβ-1 rabbit polyclonals, or anti-CDK2 and anti-VEGF-A (SC7269) mouse monoclonals from Santa Cruz Biotechnology; and anti-PARP/cPARP rabbit monoclonal (#9532) from Cell Signaling. CDK inhibitors including SNS (SNS-032), JNJ (JNJ7706621), DIA (Dinaciclib, SCH727965), PD (Palbociclib, PD0332991) and BMS (BMS-265246) were commercially obtained from Selleckchem, except for CYC065 which was kindly provided by Cyclacel (Dundee, UK). CDK inhibitors were dissolved in DMSO to produce a 10 mM stock concentration that was stored at -20 °C. ON-TARGET plus SMART pool siRNA oligonucleotides to CDK1, CDK2 and CDK9 were obtained from Dharmacon (GE Dharmacon, Lafayette, CO) and were transfected into MCF7 cells using Lipofectamine 2000 (ThermoFisher Scientific).

### MCF7 cell expression of Y537S and D538G mutated ERα

MCF7 sublines transiently or constitutively expressing ERα activating mutations (Y537S or D538G) were produced by either Lipofectamine transfection or *ESR1* gene targeting (knock-in). For transient ectopic expression, MCF7 cells were lipofectamine transfected with either a wildtype ERα construct (wt: pSG5-HEGO), or pSG5-HEGO constructs modified to express either Y537S (#37) or D538G (#38) mutations in the ERα DNA-binding domain (exon 10) using sequence verified plasmids and our previously described approach [11]; 24 h after culture transfection, cells were exposed to charcoal stripped media for an additional 24-48 h followed by cell lysate preparation for IP and IB. *ESR1* MCF7mutER knock-in sublines constitutively expressing either the Y537S or D538G mutation were produced using recombinant AAV vectors as previous described [42]. In brief, infectious AAV viral vectors harboring either the *ESR1* Y537S or the D538G mutation were prepared in HEK-293T cells with approximately 10^6^ MCF7 cells used for each viral infection. Neomycin resistant clones were isolated and screened via a modified PCR strategy then exposed to Cre-expressing recombinant adenovirus to remove the neomycin cassette [43]. All isolated clones were confirmed by Sanger sequencing and droplet digital PCR of genomic DNA and cDNA to ensure the clones harbored the intended *ESR1* knock-in mutation as a single copy with expression equal to the remaining wild type *ESR1* allele. Primer sequences for PCR amplification, mutagenesis, targeting and sequencing are shown in Supplement Table S1.

### Tumorigenicity and treatment of xenografted MCF7mutER tumors

Animal studies reported here (PTC1797, PTC1854) were conducted under IACUC approval (AN092211) in the UCSF Cancer Center's Preclinical Therapeutics Core (PTC). MCF7mutER (Y537S, D538G, AAV controls) knock-in sublines were first expanded *in vitro* and then subcutaneously (sc) injected to evaluate xenograft tumor growth. In brief, NCR nu/nu athymic female mice (6 weeks old; Taconic Farms, Germantown, NY) were implanted with 1×10^7^ MCF7mutER cells sc in the upper back area as a 0.1 mL suspension in serum free media 1:1 with matrigel. Tumor growth was measured by caliper along the largest (length) and smallest (width) axes as well as body weights were determined twice a week. Tumor volumes were calculated using the following formula: tumor volume = [(length) x (width) x (width)] / 2. Approximately 8 days after tumor implantation (mean tumor volume = 100 mm^3^) animals were randomized into two primary treatment groups (Tamoxifen vs. vehicle), and approximately 14 days after first treatment initiation (study day ~34, when mean tumor volumes of vehicle and single agent treated mice reached ~300 mm^3^), mice were further divided into additional treatment groups as indicated (+/- Dinaciclib, +/- Palbociclib), and these secondary treatments carried for ~14 days. Tamoxifen (TAM) citrate was prepared in a 10 mg/ml suspension in peanut oil vehicle and either TAM (0.5 mg) or vehicle alone was delivered sc daily as previously studied [44, 45]. Dinaciclib (30 mg/kg), Palbociclib (30 mg/kg), or vehicle (20% hydroxypropyl-b-cyclodextrin) was delivered by intraperitoneal injection (ip) every 3 days on the indicated study days, dosing based on prior studies [46, 47] and preliminary experiments establishing *in vivo* tolerance of tumor-bearing mice (< 10% loss of body weight) to repeated ip Dinaciclib doses when given in combination with daily sc TAM. At study conclusion, all animals were euthanized and tumors resected and snap frozen (-| || || |C) within 2-3 h following the final treatment dose.

### Tumor cell lysates, protein immunoprecipitation and immunoblotting

Monolayer cell cultures were harvested in ice cold lysis buffer [100 mmol/L NaCl, 20 mmol/L Tris (pH 7.5), 0.5% IGEPAL-630, 1 tablet/10 mL PhosSTOP (Roche Applied Science), 320 nmol/L okadaic acid, and 1 tablet/10 mL Roche mini-complete protease inhibitor cocktail (Roche Applied Science)]; tumors and xenografts were first pulverized under liquid nitrogen and then sonicated in lysis buffer. To prepare isolated nuclei, cells were first lysed in low salt buffer (10 mM Tris pH 7.5, 50 mM NaCl, 0.4% NP40 plus above protease and phosphatase inhibitors; pelleted cell free nuclei were resuspended in nuclear extraction buffer (0.4 M KCl, 20 mM Hepes pH 7.5, 1.5 mM MgCl_2_ and 10% glycerol) for 20 min and residual chromatin removed by centrifugation. Protein from the resulting cleared supernatant was IP by the addition of 8 μl of F-10 anti-ERα antibody (at 0.2 mg/ml) with 15 μL Protein G Sepharose 4 Fast Flow beads (GE Healthcare) and incubated with slow rotation at 4°C for 6 h. Beads were then pelleted washed 3 times in wash buffer (125 mmol/L NaCl, 20 mmol/L Tris, pH 7.5, and 0.35% IGEPAL). IP samples were then suspended in Laemmli loading buffer and analyzed by IB as previously described [11]. Immunoblot film images were scanned and imported into a ChemiDoc XRS system (Bio-Rad), and utilizing the ChemiDoc analysis software the area around each immunoblot band of interest was selected and quantified, with the intensity of each band minus the background intensity used to quantify protein levels.

### Semiquantitative reverse-transcription PCR

As previously described [11], total RNA was harvested using Trizol followed by treatment with DNA-free (Ambion, Austin, TX) according to the manufacturer's specifications to remove potentially contaminating DNA. Reversed transcription was performed using oligo dT priming of 0.5 μg RNA per sample condition with SuperScript II (Invitrogen, Carlsbad, CA) according to manufacturer's specifications. PCR reactions used 1 μl aliquots from the RT reactions with Pfu polymerase (New England Biolabs, Ipswich, MA). Reaction conditions consisted of annealing at 60°C for 30 sec, extension at 72°C for 25 sec and denaturation at 96°C for 10 sec with identically prepared reactions subjected to 24, 26 or 28 PCR cycles. PCR products were electrophoresed on 8% polyacrylamide gels, stained with eithidum bromide, photographed and quantified by densitometry using a GS-710 Calibrated Imaging Densitometer (Bio-Rad, Hercules, CA). Primers included: AREG (Amphiregulin) 170bp 5’aaaaagggaggcaaaaatgg3’ (forward), 5’tcatggacttttccccaca3’ (reverse); EGR3 238bp 5’gcagcatggtcttgactgaa3’ (forward), 5’ccccctttccactagagtcc3’ (reverse); CXCL12 221bp 5’ctagtcaagtgcgtccacga3’ (forward) 5’ggacacaccacagcacaaac3’ (reverse); GAPDH 234bp 5’cgaatttggctacagcaacagg3’ (forward), 5’gtacatgacaaggtgcggctc3’ (reverse).

## SUPPLEMENTARY MATERIALS FIGURES AND TABLES





## Abbreviations

ERα: estrogen receptor alpha
ERmut: mutationally activated ERα
CDK: cyclin-dependent kinase
ERpS294 (or pS294): ERα phosphorylation at serine-294
