# Supplementary material for: ERpS294 is a biomarker of ligand or mutational ERα activation and a breast cancer target for CDK2 inhibition
Source: Oncotarget. 2016 Oct 18;8(48):83432–45. doi: 10.18632/oncotarget.12735 (PMC5663526; doi:10.18632/oncotarget.12735)
Supplement: Supplementary file 1 [file oncotarget-08-83432-s001.pdf]

ERpS294 is a biomarker of ligand or mutational ERα activation and a breast cancer target for CDK2 inhibition

Supplementary Material

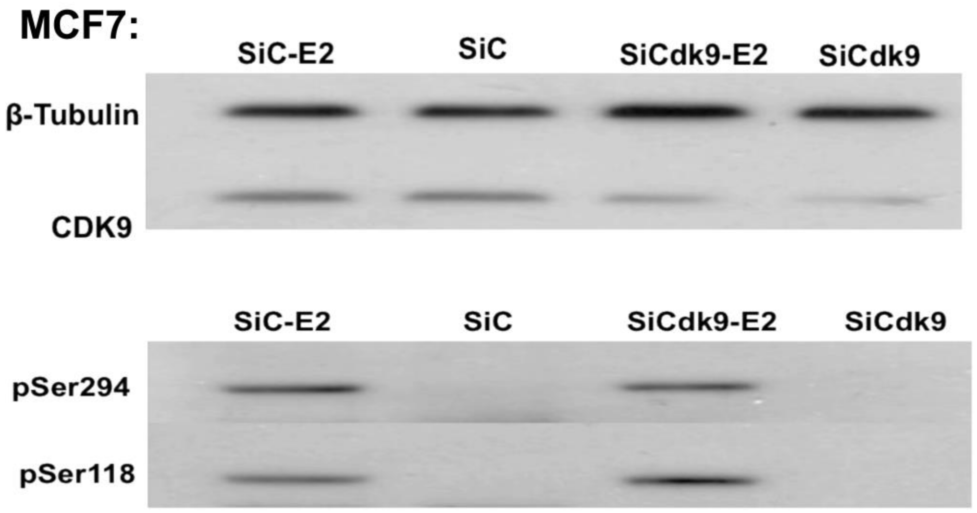

Supplement Figure S1. Knockdown of the non-mitotic CDK9 target of Dinaciclib has no impact on E2 induction of pS294 in MCF7 cells. As described in Figure 2, replicate wells of MCF7 cells were transiently transfected with either control (C) or CDK9 targeted siRNA; 24 h later cultures were changed to phenol red-free media containing 10% charcoal-stripped serum and allowed to grow for another 24 h before stimulation by E2 (10 nM x 20 min), followed by cell harvesting, ERα immunoprecipitation and immunoblotting for pS294 or pS118. Immunoblot densitometry confirms that knockdown of CDK9 produced minimal effect on ligand induced ERα phosphorylation: E2 induction of pS294 = 1.38 (siCdk9-E2 band intensity/siC-E2 band intensity), comparable to the E2 induction of pS118 = 1.45.

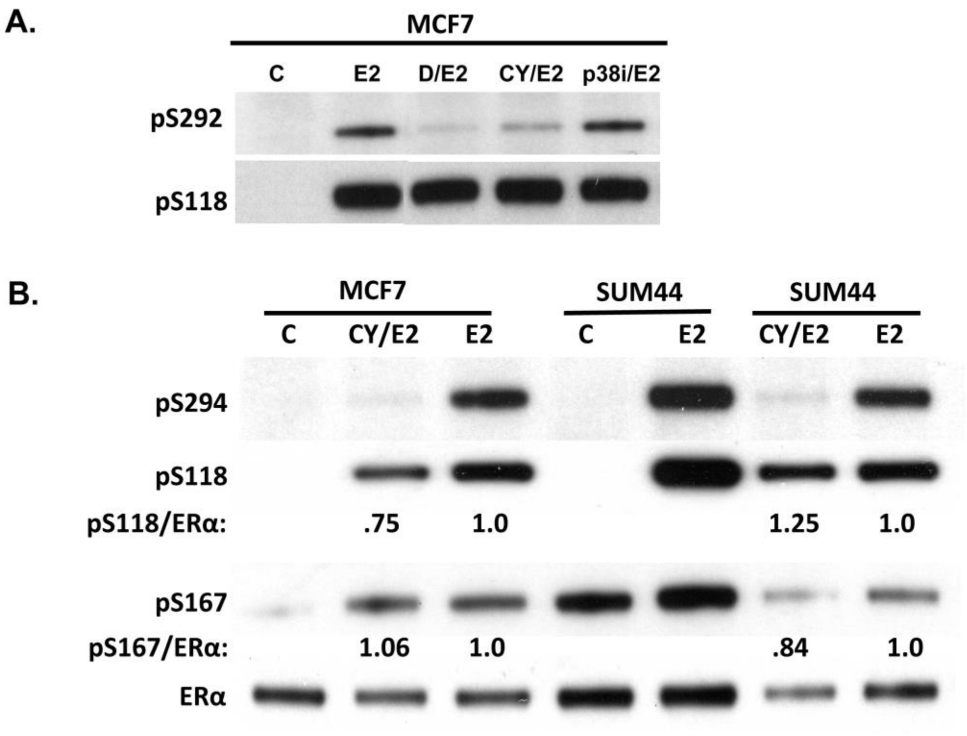

Supplement Figure S2. CDK2 inhibitors, but not a p38 inhibitor, selectively prevent pS294 induction in different ER-positive breast cancer cell line models (MCF7, SUM44). Control cells (C) were grown in phenol red-free media supplemented with 5% charcoal stripped serum for 24 h; before estradiol (E2, 10 nM x 20 min) stimulation, some were pretreated with either the CDK1/2/5/9 inhibitor Dinaciclib (D, 0.5 μM x 30 min in **A**), the CDK2/9 inhibitor CYC065 (CY, 0.5 μM x 30 min in **A** or 1.0 μM x 20 min in **B**), or the p38 inhibitor SB203580 (p38i, 1.0 μM x 30 min in **A**). After E2 stimulation cells were harvested, protein extracted, total ERα immunoprecipitated and then immunoblotted for pS294, pS118, pS167 or total ERα. Like MCF7 cells, SUM44 cells showed E2 induction of pS294 prevented by CY, and E2 induction of pS118 which was largely unaffected by CY pretreatment. Unlike MCF7 cells, SUM44 cells showed constitutive basal expression of pS167, which remained largely unaffected by CY pretreatment (**B**).

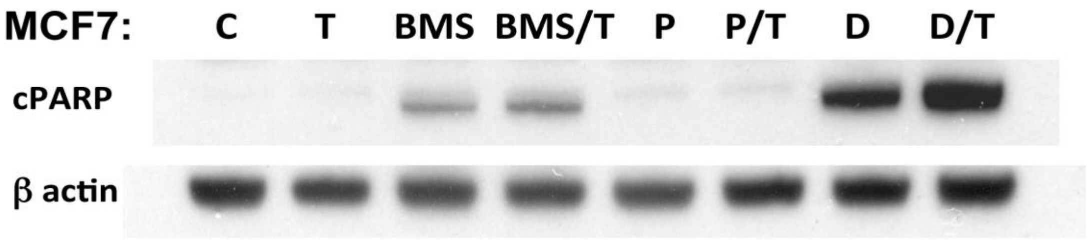

Supplement Figure S3. CDK2 inhibitors, but not a CDK4/6 inhibitor, cooperate with tamoxifen to induce ER-positive breast cancer cell apoptosis. MCF7 cell cultures at mid-confluency were treated with vehicle (C) or 4-hydroxytamoxifen (T, 1.5 μM x 48 h); during the final 6 h before harvest cultures were treated with either BMS-265246 (BMS, 1 μM x 6 h), Palbociclib (P, 1 μM x 6 h) or Dinaciclib (D, 0.5 μM x 6 h). Cell lysates were protein extracted and then immunoblotted for cleaved (c) PARP and β actin.

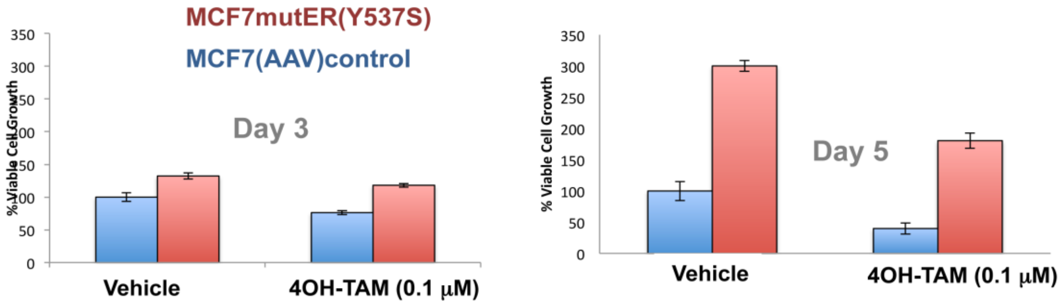

Supplement Figure S4. MCF7 cells overexpressing mutated ERα, MCF7mutER(Y537S), show more rapid and tamoxifen-resistant growth in vitro than MCF7(AAV)control cells. The knock-in sublines of MCF7, passaged and plated into replicate wells with standard serum-containing media, were then switched to phenol red-free media supplemented with 5% charcoal stripped serum (day 0) and 24 h later exposed to either vehicle or 4-hydroxytamoxifen (4OH-TAM, 0.1 μM). Cell viability was assessed at days 3 and 5, and growth scored relative to that of vehicle treated MCF7(AAV)control cells (100%) for each time point. tamoxifen significantly prevented MCF7(AAV)control cell growth, moreso by 5 days; in contrast, MCF7mutER(Y537S) cells continued to grow more rapidly than vehicle treated control cells for 5 days despite 4OH-TAM exposure, although by day 5 the vehicle treated MCF7mutER(Y537S) had outgrown the 4OH-TAM exposed MCF7mutER(Y537S) cells.

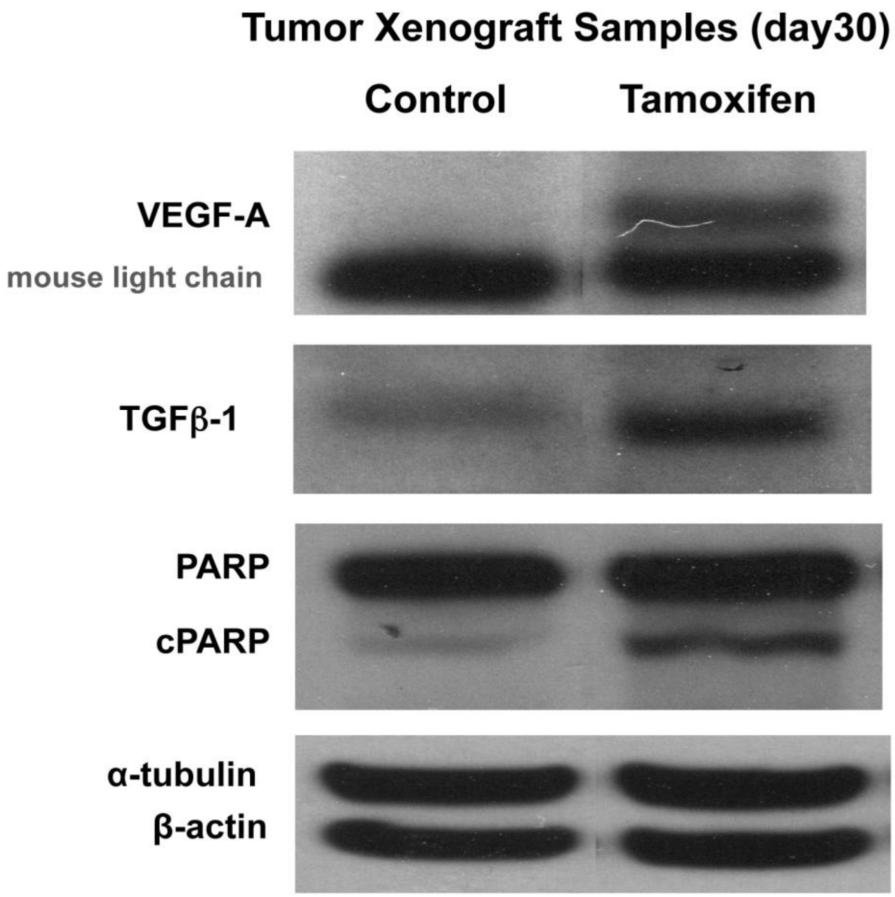

Supplement Figure S5. Tamoxifen effects on xenografted MCF7mutER(Y537S) tumors from study PTC1854. MCF7mutER(Y537S) xenografted nude mouse tumors (growing without E2 supplementation) were treated daily as described in Figure 4B, beginning at day 8; enlarging Control (vehicle) and tamoxifen treated tumors from PTC1854 were excised and snap frozen at study day 30. Tumor lysates, after first being immunoprecipitated for pS294 and then total ERα, were immunoblotted for VEGF-A, TGFβ-1, PARP and cleaved (c) PARP, α-tubulin and β-actin to demonstrate apparent tamoxifen treatment effects despite continuing tumor growth. The partially obscuring mouse light chain band is due to the use of a secondary goat anti-mouse system to image the tumor's VEGF-A band.
